# Pediatric Prostatic Abscess Caused by Methicillin-Resistant Staphylococcus aureus

**DOI:** 10.7759/cureus.20598

**Published:** 2021-12-22

**Authors:** Atef A Rashed, Moath H Albarakati, Ola G Fintyana, Ruwaidah H Melebary, Albandari H Alharbi, Deemah H Bukhari, Asem A Rashed

**Affiliations:** 1 General Pediatric, Pediatric Department, Maternity and Children's Hospital, Makkah, SAU; 2 Family Medicine, Maternity and Children's Hospital, Makkah, SAU; 3 Medicine, Umm Al-Qura University, Makkah, SAU; 4 Otolaryngology - Head and Neck Surgery, Maternity and Children's Hospital, Makkah, SAU; 5 Medicine, Pediatric Department, Maternity and Children's Hospital, Makkah, SAU

**Keywords:** methicillin-resistant staphylococcus aureus (mrsa), ultrasound-guided aspiration, abscess, prostate, pediatric

## Abstract

While prostatic abscesses infrequently occur in adults, they are extremely rare in children. We present a rare case of a prostatic abscess in a 13-year-old male patient caused by methicillin-resistant *Staphylococcus aureus* (MRSA). The patient had no significant past history and presented to our clinic reporting a two-week history of lower abdominal pain, foul-smelling urethral discharge, a burning sensation during urination with pain in the tip of his penis, and itchiness around the anus. On examination, we noted lower abdominal tenderness, and on per rectal examination, we noted tenderness in the anterior wall of the rectum. A culture from the urethral discharge was positive for MRSA. The patient was diagnosed with a prostatic abscess and was started on antibiotics. We performed ultrasound-guided transrectal drainage, and afterward, the patient’s condition improved. He was doing well on the last follow-up. This case reminds physicians to consider prostatic abscesses in patients with lower urinary tract infections that do not respond to antibiotics.

## Introduction

A prostatic abscess is a rare condition, especially in the pediatric population. Very few cases of pediatric prostatic abscess attributed to methicillin-resistant *Staphylococcus aureus* (MRSA) have been documented in the literature. *Staphylococcus aureus* is a rare cause of prostatic abscess, and gonorrhea was the most common etiology prior to using antibiotic medications. However, with the advantages of antibiotic use, Gram-negative bacteria are the most common organisms causing prostatic abscesses and are responsible for 60%-80% of cases [1]. Surgical drainage with adequate antibiotics is the recently recommended management for prostatic abscesses larger than 1 cm. However, the appropriate management of a prostatic abscess remains questionable [2].

## Case presentation

A 13-year-old male with no previous medical history presented to our clinic reporting a lower abdominal pain lasting two weeks. He also reported experiencing a foul-smelling urethral discharge and burning sensation on urination, with pain at the tip of his penis. He had no history of fever, sexual activity, hematuria, or back pain, and he mentioned itchiness around his anus. we noted he had visited the emergency department one week prior with the same reported concerns. On examination, the patient looked well, his vitals were stable, and he is afebrile, with normal activity and interactions. An examination of his lower abdomen revealed abdominal tenderness, and a rectal examination revealed tenderness in the anterior wall of the rectum. His scrotum examination revealed Tanner stage 5 anatomy with no other remarkable findings.

His initial laboratory workup results were within the reference ranges, chlamydia and gonorrhea were negative, and his serology tests for human immunodeficiency virus, hepatitis B virus, and hepatitis C virus were nonreactive. The second and third urine cultures showed no growth, and his blood culture showed no growth. The urethral discharge sample culture was positive for MRSA. The patient was admitted to the pediatric department as a case of urinary tract infection, and we started him on ceftriaxone and clindamycin to rule out any sexually transmitted diseases.

**Figure 1:**
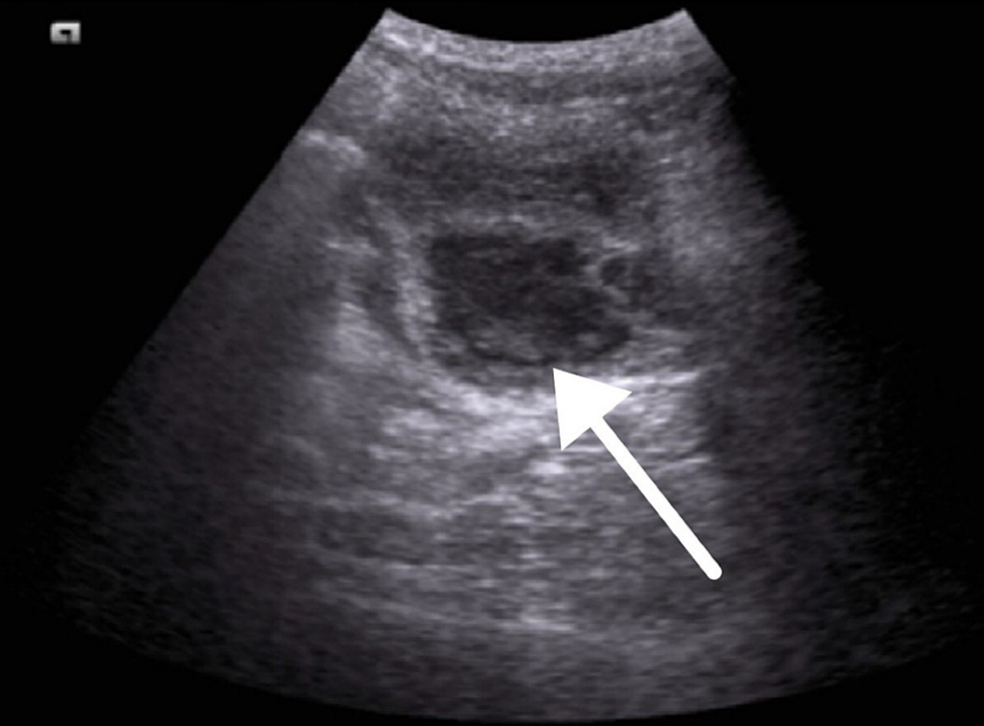
Ultrasound of the bladder revealing a well-defined, thick-walled hypoechoic lesion posterior to the urinary bladder measuring 5.3 × 3.7 cm

We conducted ultrasonography for the kidney and bladder and incidentally noted a small amount of free fluid in the pelvis. We also noted a well-defined, thick-walled hypoechoic lesion posterior to the urinary bladder measuring 5.3 × 3.7 cm (Figure 1).

**Figure 2:**
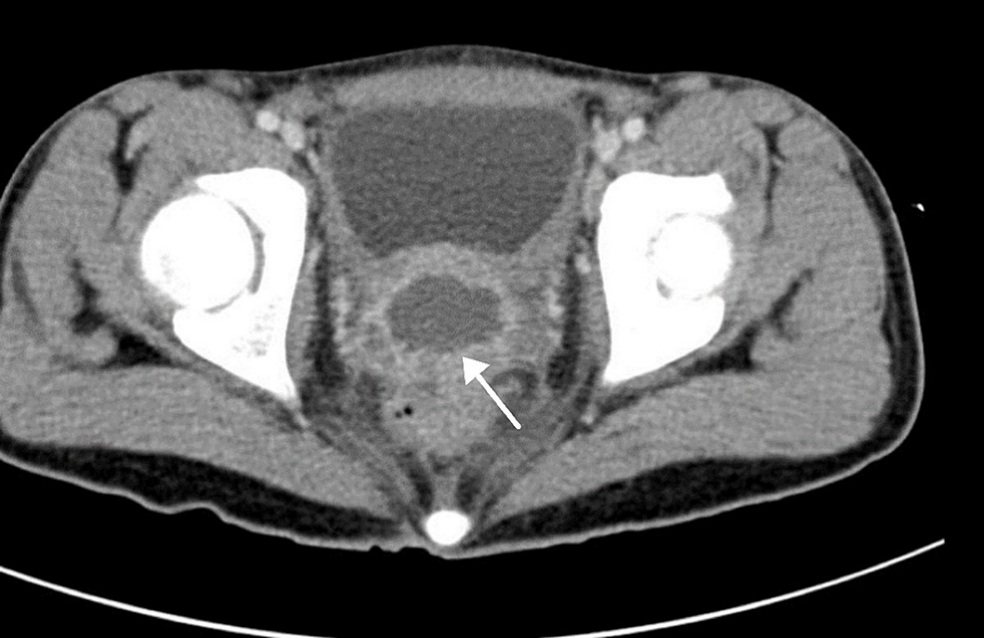
Computed tomography (axial view) revealing multilocular and septated prostatic cyclic lesion measuring approximately 5 × 4 × 3 cm that could be a cystadenoma or prostatic abscess

**Figure 3:**
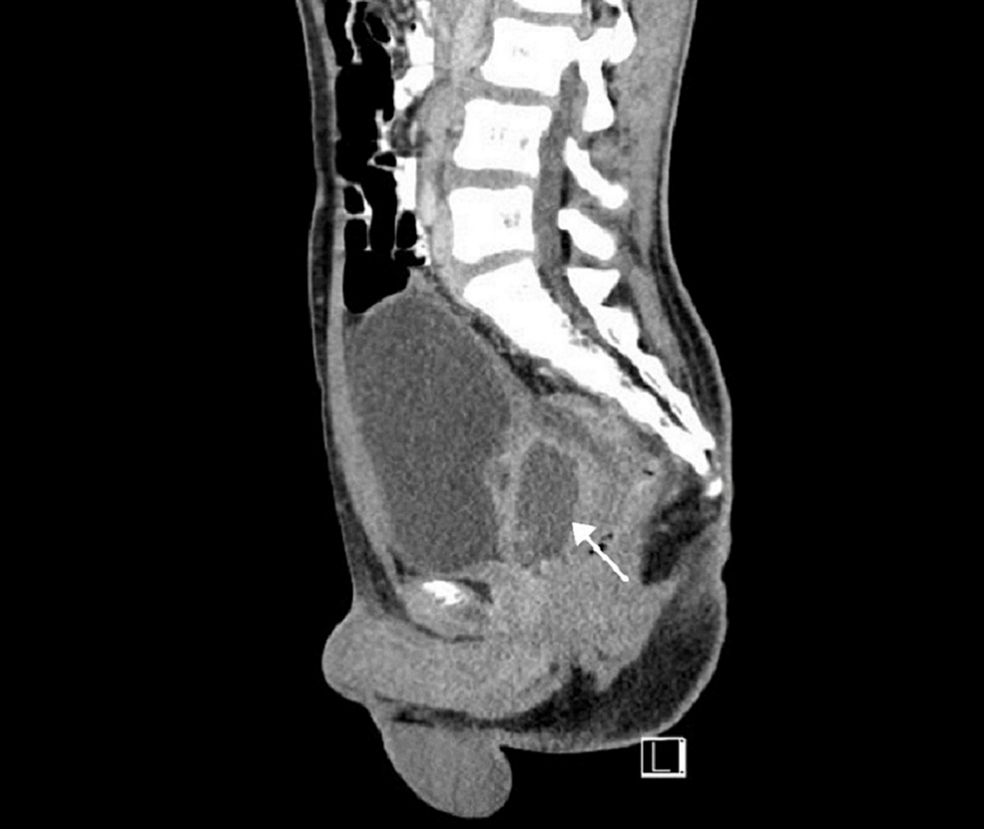
Computed tomography (sagittal view) revealing cystic lesion compressing on the posterior bladder wall

A computed tomography (CT) showed evidence of a multilocular and septated prostatic cystic lesion measuring approximately 5 × 4 × 3 cm, which could be a cystadenoma or prostatic abscess to be confirmed by a histopathological examination (Figure 2 and Figure 3).

We performed an ultrasound-guided aspiration that released bloody pus. A cytology investigation showed mostly neutrophils with no malignant cells. The prostatic abscess diagnosis was confirmed. The patient was kept on ceftriaxone and clindamycin antibiotics. He improved gradually but started to have bloody discharge from the urethra even with the improvement of other symptoms. Later, however, all his symptoms improved, and he was doing well as of this writing.

## Discussion

The diagnosis of a prostatic abscess is often challenging, and persistent symptoms despite antibiotic treatment should raise suspicions for this condition [3].

The symptoms overlap with symptoms of other lower urinary tract diseases, and prostatic abscesses are often diagnosed after patients’ conditions are unresponsive to initial medical treatment. Rectal examination is not routine for the pediatric age group, although rectal examinations can lead to a correct diagnosis. The presence of urethral discharge, common urinary symptoms, and unresponsiveness to antibiotic treatment should point clinicians to the possibility of a prostatic abscess [1].

Fistulation to the surrounding pelvic structures may complicate prostatic abscesses. Abscesses located at the base of the prostate gland commonly fistulate to the bladder, prostatic urethra, or rectal or perianal spaces. Severe cases of a prostatic abscess may extend into the seminal vesicle and spermatic cord [4]. Early diagnosis is essential to avoid complications. CT scans and ultrasonography are necessary diagnostic tools for patients with this condition. The surgical treatment of prostatic abscess includes transperineal or transurethral drainage with antibiotic therapy guided by culture results [5]. Prostatic abscesses are commonly due to *Escherichia coli* and other Gram-negative bacteria - very rarely is it caused by *Staphylococcus aureus* [6,7]. Reports of prostatic abscesses because of MRSA have increased recently [8-10]. Patients with diabetes, immunocompromised state, and urinary tract abnormalities are at higher risk for prostatic abscess than otherwise healthy patients. Our patient was infected by MRSA, a rare cause of a prostatic abscess, and he had none of the common risk factors for MRSA, such as prolonged hospital stays or a compromised immune system. We could not determine the patient’s sexual status in taking his medical history, but physical and laboratory examinations ruled out sexually transmitted disease.

## Conclusions

A prostatic abscess can be a diagnostic and therapeutic challenge. We discussed a rare case of prostatic abscess secondary to MRSA infection in a 13-year-old male. Our case highlights the importance of considering prostatic abscess as the cause of urinary tract symptoms and the importance of rectal examination in any patient with lower urinary tract symptoms - especially in patients with urethral discharge or those who do not respond to antibiotic treatment.
